# Nutritional treatment of aged individuals with Alzheimer disease living at home with their spouses: study protocol for a randomized controlled trial

**DOI:** 10.1186/1745-6215-13-66

**Published:** 2012-05-24

**Authors:** Satu K Jyvakorpi, Taija Puranen, Kaisu H Pitkala, Merja H Suominen

**Affiliations:** 1 Society for Memory Disorders Expertise in Finland, Fredriksberginkatu 2, 00240, Helsinki, Finland; 2 Unit of General Practice, Helsinki University Central Hospital and Department of General Practice and Primary Health Care, University of Helsinki, PO Box 20, 00014, Helsinki, Finland

**Keywords:** Nutritional intervention, Malnutrition, Alzheimer disease, RCT, Nutrient intake

## Abstract

**Background:**

Nutritional status often deteriorates in Alzheimer’s disease (AD). Less is known about whether nutritional care reverses malnutrition and its harmful consequences in AD. The aim of this study is to examine whether individualized nutritional care has an effect on weight, nutrition, health, physical functioning, and quality of life in older individuals with AD and their spouses living at home.

**Methods:**

AD patients and their spouses (aged >65 years) living at home (n = 202, 102 AD patients) were recruited using central AD registers in Finland. The couples were randomized into intervention and control groups. A trained nutritionist visited intervention couples 4–8 times at their homes and the couples received tailored nutritional care. When necessary, the couples were given protein and nutrient-enriched complementary drinks. All intervention couples were advised to take vitamin D 20 μg/day. The intervention lasted for one year. The couples of the control group received a written guide on nutrition of older people. Participants in the intervention group were assessed every three months. The primary outcome measure is weight change. Secondary measures are the intake of energy, protein, and other nutrients, nutritional status, cognition, caregiver’s burden, depression, health related quality of life and grip strength.

**Discussion:**

This study provides data on whether tailored nutritional care is beneficial to home-dwelling AD patients and their spouses.

**Trial registration:**

ACTRN 12611000018910

## Background

Older individuals with Alzheimer disease (AD) living at home with their spouses are an important target group for nutritional care [1] because weight loss and malnutrition are common problem among them [2-4]. Weight loss is a predictive factor of mortality, and it decreases AD patients' and caregivers' quality of life (QOL) [5,6]. Furthermore AD patients often suffer from frailty, sarcopenia, functional impairments, and comorbidities [7]. Their old spousal caregivers often also have comorbidities and functional disabilities [8]. The possibilities of nutritional care in this patient group remain largely unexplored [9].

Nutritional interventions may have beneficial effects on nutritional status and nutrient intake of AD patients [6,10-13]. Several controlled studies have suggested that nutritional supplements increase body weight of AD patients at risk of malnutrition. In an intervention study, weight gain of AD patients was achieved when nutritional supplements were taken for three months [14]. Similarly, Lauque et al. [10] showed that AD patients’ body weight significantly increased with oral nutritional supplements given for three months. A nutrition educational program directed at AD patients and their spouses had a positive effect on AD patients’ weights and their cognitive function [6,13].

However, further studies on the benefits of nutritional care and ways of implementing it are needed [15]. Nutritional care and tailored nutritional counselling are important and largely unexplored means of maintaining the health and QOL of AD patients and their caregivers.

Although nutritional care has been examined and proven effective in randomized, controlled settings in a few multinational studies [1,6,13], the effects of nutritional care on functional ability, health, QOL and use of health services among the aged have not been investigated.

The aim of this study is to investigate whether tailored individualized nutritional counselling and care have an effect on the weight, nutrition, functioning, and QOL of AD patients and their spouses.

## Methods

### General design

This is an intervention trial with a randomized design. The intervention lasts for one year. The intervention group is compared with the control group, who have received a written guide on nutrition of older people. The study has been approved by the Ethics Committee of the Helsinki University Central Hospital and the research committee of KELA (The Social Insurance Institution of Finland). Informed consent has been obtained from each participant and their spousal caregiver before any study procedure.

### Participants

In 2010, a sample of AD patients and their spouses living at the same address in the Vantaa area of Finland was retrieved from the centralized Drug Imbursement Register of the Social Insurance Institution of Finland (KELA). All patients had undergone detailed diagnostic assessments (e.g. cognitive and neuropsychological tests, neuroimaging, and laboratory tests), before receiving reimbursement for their AD medication. The first recruiting letter was sent by KELA with a reply form. Investigators then contacted those who replied. The study purpose and protocol were disclosed to the participants and they were invited to participate. The inclusion criteria were as follows:

 A person with AD living with an aged spouse

 age over 64 years

 being able to use transport (arrive to the study place by taxi)

 being able to stand on a scale

 living in the greater Helsinki area (Helsinki-Espoo-Vantaa)

 adequacy in the Finnish language

 no terminal disease

All AD patients and their spouses showing interest in participation were first contacted and interviewed by phone to confirm the fulfillment of the inclusion criteria. Those couples fulfilling the criteria were invited to the first meeting with a nutritionist, where they were given oral and written information about the study and asked to sign an informed consent. In case of an AD patient’s inability to give informed consent, the spousal caregiver gave proxy consent for both spouses.

### Measurements

The baseline measurements include: background information about demographic data, diagnosis, other potential diseases, falls, fractures, and medication. This information is confirmed from medical records provided by the couples at the first meeting.

The first meeting with baseline measurements takes about two hours. The participants’ (AD patient and spouse) weight, height, and grip strength are measured and body mass index (BMI) is calculated. Weight is measured with a portable, calibrated scale (Dexa).

Both spouses’ nutritional status is assessed by using Mini nutritional Assessment (MNA) [16] and nutritional anamnesis. In addition, the AD patient and the spouse are both assessed by Minimental State Examination (MMSE) [17]. Health-related quality of life (HRQOL) is measured with 15D- HRQOL form [18] and caregiver’s burden with the ZARIT form [19].

The AD patients are assessed by Clinical Dementia Rating Scale (CDR) [20], Neuropsychiatric Inventory (NPI) [21,22], Cornell depression test [23], and Instrumental activities of daily living (IADL) [24] by interviewing the spouse. The spouse is also assessed by IADL. While the spouse is being interviewed, the AD patient has the opportunity to take part in day-time group activities for older people provided by the city of Vantaa in Simonkylä.

A trained nutritionist gives AD patients’ spouses oral and written instructions on how to keep a food diary for three days. They receive food measures of 100 ml, 15 ml, and 5 ml to measure amounts of food items consumed. After investigators receive the completed food diary, the nutritionist checks it and calls the couple to ensure that the amounts of food items, cooking methods, and, for example the type of milk or fat are recorded correctly. The food diaries are analyzed using a Nutrica 3.11 [25] program developed for this purpose.

### Randomization

The couples are randomized into clusters of six. The randomization is performed as follows: investigators write down identification numbers of each couple on six pieces of papers, which are then folded so that the numbers cannot be seen. A person unrelated to the investigation then chooses three pieces of paper and the couples with these ID numbers are signed to the intervention group.

All participating couples are assessed at baseline and at the end of the trial with methods described previously. In addition, all AD patients’ and their spouses’ weights are measured at six months.

The nutritionist visits and weighs the intervention couples in their homes also at 3 and 9 months. Nutritional counseling and care are tailored according to each intervention couples needs and consists of 4–8 home visits by the nutritionist Table 1.

**Table 1:** Study assessments and timetable

**Assessment**	**Baseline assessments**	**Telephone interview**	**3-month visit**	**6-month visit**	**9-month visit**	**12-month assessment**
**AD patient**						
Inclusion criteria		x				
Background information^1^	x					x
Nutrition anamnesis	x					x
3-day food diaries^1^	x	x				x
CDR	x					x
MMSE	x					x
MNA	x					x
Weight, BMI	x		x^2^	x	x^2^	x
Grip strength	x					x
NPI	x					x
Cornell	x					x
IADL	x					x
15 D^1^	x					x
Use of health services				x		x
**Spouse**						
Background information^1^	x					x
Nutrition anamnesis	x					x
3-day food diaries^1^	x	x				x
Zarit^1^	x					x
MNA	x					x
MMSE	x					x
15 D HRQOL^1^	x					
IADL	x					x
Weight, BMI	x		x^2^	x	x^2^	x
Grip strength	x					x
Use of health services				x		x

^1^filled at home.

^2^only in intervention group.

*AD *Alzheimer’s disease, *CDR *degree of dementia [20], *MMSE *Mini mental state examination [17], *MNA *Mini Nutritional Assessment [16], *BMI *body mass index, *NPI *Neuropsychiatric inventory [21,22], *Cornell *depression test [23], *IADL *Instrumental activities of daily living [24], *15 D *HRQOF [18], *Zarit *Zarit burden scale [19].

### Intervention

The nutritionist analyzes the 3-day food diaries recorded by the couples. On the basis of the food diary analysis, MNA, home visits and nutritional anamnesis, the nutritionist develops a plan for individualized nutritional care and discusses nutrition with the couple.

The objective of nutritional care is not to change the AD patients’ and spouses’ food habits completely, but to correct possible inadequacies in their diet. The main objective is to recommend food items that they are familiar with and which are already part of their daily diet. If nutrient deficiencies on the basis of food diaries or further discussions with the spouse are noted that are difficult to correct with food, a supplement is recommended. Nutritional care has the objective of ensuring a sufficient intake of energy, protein, and other nutrients. To increase protein in the diet, good protein sources are recommended, and if the couple seems unable to increase energy, protein, and/or other nutrients in their diet, complementary dietary drinks consisting of protein and other nutrients are given to them for daily use. The complementary drink [Nutridrink protein (200 ml; 300 kcal, 20 g protein) or Nutridrink compact (125 ml; 300 kcal and 12 g protein)] is offered once daily.

**Figure 1:**
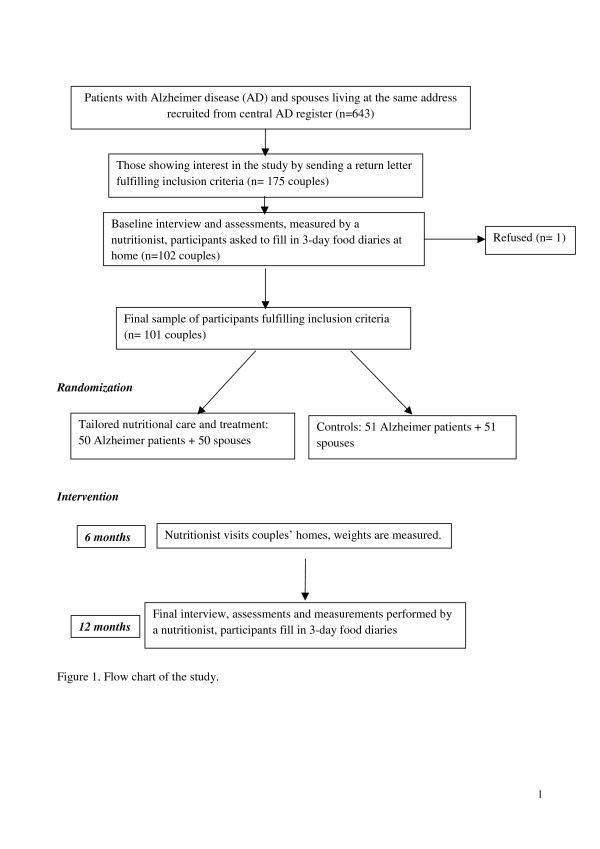
Flow chart of the study.

All intervention couples are also advised to take 20 μg of supplemental vitamin D daily. The amount of calcium in the diets of participants is also reviewed. The nutritionist visits the couples at least four times and weighs them with a portable scale at 3, 6, and 9 months. At baseline and at the final meeting at 12 months, the weights are also measured along with other assessments. The nutritionist can visit couples’ homes more frequently according to their needs. The intervention couples also receive oral and written advice on exercise to strengthen muscles that is easy to perform at home. The intervention lasts for one year (Figure 1).

In addition to the nutritional counseling, the couples in the intervention group have the opportunity to take part in a group meeting discussing nutritional issues. Couples willing to participate receive a free taxi-card to arrive to and from the meeting.

### Outcome

A primary outcome measure is change in AD patients’ weights. Secondary outcomes are changes in their energy, protein, and other nutrient intakes retrieved from food diaries, AD patients’ functional ability [20,24], cognitive functioning (MMSE) [19], neuropsychiatric symptoms (NPI, Cornell) [21-23], and caregiver burden (Zarit burden scale) [17]. In addition, HRQOL [16] and use of health services will be assessed during the on year follow-up.

### Statistical analyses

Sample size is calculated based on AD patient’s expected weight change, with a standard deviation of 3.6 kg [6], and type I error of 5%, 80% power. For an expected weight difference between intervention and control patients of 2 kg, 50 patients are needed in each group to show statistical difference. Data will be analyzed on an intention to treat basis. Imputation methods of “the last observation carried forward” (LOCF) and worst rank score will be used.

Data are analyzed using a SPSS statistical program. Intervention and control groups will be compared at baseline; categorical variables will be tested by a Chi-squared test (X^2^) or Fischer’s exact test, and continuous variables by two-sided t-test or Mann–Whitney U-test as appropriate.

Qualitative data will be collected by interviewing subjects during the nutritionist’s visits. The case studies will be transcribed and content analyses carried out. The aim of the analyses will be to analyze difficulties and positive factors in the daily lives of the families that may explain differences in success of nutritional care between subjects.

All data will be securely stored at The Society for Memory Disorders Expertise in Finland. Computerized data will be available only to the members of the research team. The analyzed data will be cleared of all the recognition information, and only ID numbers will be used.

## Discussion

This intervention trial investigates the effect of individualized, home-based tailored nutritional care on home-dwelling AD patients and their aged spouses. A nutritionist visits the intervention couples in their homes 4–8 times according to their nutritional needs. In addition, the intervention group is given oral and written counseling on exercise and how to perform daily exercise routine to strengthen muscles. If the intervention couple is unable to complement their diet naturally and clear deficiencies in protein and/or other nutrients are observed, the couple will be given complementary daily drinks that contain energy, protein, and other nutrients. Dietary supplements are recommended to AD patient and spouse when necessary. In addition, intervention couples are advised to take a 20 μg vitamin D supplement daily year round. The control group is given a written guide on nutrition of the older people.

Our study has several strengths. All AD patients have an established diagnosis of AD; they were recruited from the Finnish drug registry. As AD is a risk factor for deterioration of nutritional status, this intervention enables this problem to be dealt with at early stage of the disease. Old spouses also often have multiple diseases and disabilities, and thus, are an important target group for nutritional care. Tailored nutritional care may enable the aged couple to live in their home longer. Findings of this trial are directly applicable to real life, allowing nutritionists and healthcare professionals to focus on nutritional interventions for the older people. Tailored nutritional intervention that addresses the nutritional needs of AD patients and their caregivers is described in detail.

The study also has potential drawbacks. First, our study population is old and frail with many comorbidities, thus, being vulnerable to various complications. Second, a sufficient difference may be difficult to obtain between intervention and control groups. Contamination of the control group is probably not a problem because home-based nutritional interventions are not available in Finland to AD patients and their spouses.

To our knowledge, this study is the first of its kind conducted in Finland; nutritional interventions in a home-based setting before for AD patients and their spouses have not been performed. Our study provides new and detailed information on intervention methods that can be implemented to decrease malnutrition and its adverse effects in frail old individuals. This gives caregivers and healthcare personnel information on nutritional treatment possibilities. Optimally, nutritional rehabilitation will support older persons’ nutrition, maintain or increase functional ability, reduce the use of health services, and improve QOL.

## Trial status

Trial is still ongoing. Patient recruitment has been completed and follow-up is being conducted.

## Abbreviations

AD: Alzheimer disease; BMI: Body Mass Index; CDR: Clinical Dementia Rating Scale; HRQOL: Health-related quality of life; IADL: Instrumental activities of daily living; MMSE: Minimental State Examination; MNA: Mini Nutritional assessment; NPI: Neuropsychiatric Inventory.

## Competing interests

All the authors declare that they have no competing interests.

## Authors’ contributions

1. Conception and design (SKJ, TP, KHP, MHS). 2. Acquisition of data (SKJ, TP, MHS) 3. Drafting the article or revising it critically (SKJ, TP, KHP, MHS). 4. Final approval of the version to be submitted (SKJ, TP, KHP, MHS). MHS is the guarantor.
